# Publication Bias in Recent Meta-Analyses

**DOI:** 10.1371/journal.pone.0081823

**Published:** 2013-11-27

**Authors:** Michal Kicinski

**Affiliations:** Department of Science, Hasselt University, Hasselt, Belgium; Universiteit Gent, Belgium

## Abstract

**Introduction:**

Positive results have a greater chance of being published and outcomes that are statistically significant have a greater chance of being fully reported. One consequence of research underreporting is that it may influence the sample of studies that is available for a meta-analysis. Smaller studies are often characterized by larger effects in published meta-analyses, which can be possibly explained by publication bias. We investigated the association between the statistical significance of the results and the probability of being included in recent meta-analyses.

**Methods:**

For meta-analyses of clinical trials, we defined the relative risk as the ratio of the probability of including statistically significant results favoring the treatment to the probability of including other results. For meta-analyses of other studies, we defined the relative risk as the ratio of the probability of including biologically plausible statistically significant results to the probability of including other results. We applied a Bayesian selection model for meta-analyses that included at least 30 studies and were published in four major general medical journals (BMJ, JAMA, Lancet, and PLOS Medicine) between 2008 and 2012.

**Results:**

We identified 49 meta-analyses. The estimate of the relative risk was greater than one in 42 meta-analyses, greater than two in 16 meta-analyses, greater than three in eight meta-analyses, and greater than five in four meta-analyses. In 10 out of 28 meta-analyses of clinical trials, there was strong evidence that statistically significant results favoring the treatment were more likely to be included. In 4 out of 19 meta-analyses of observational studies, there was strong evidence that plausible statistically significant outcomes had a higher probability of being included.

**Conclusions:**

Publication bias was present in a substantial proportion of large meta-analyses that were recently published in four major medical journals.

## Introduction

When some study outcomes are more likely to be published than other, the literature that is available to doctors, scientists, and policy makers provides misleading information. The tendency to decide to publish a study based on its results has been long acknowledged as a major threat to the validity of conclusions from medical research[1,2]. During the past 25 years, the phenomenon of research underreporting has been extensively investigated. It is clear that statistically significant results supporting the hypothesis of the researcher often have a greater chance of being published and fully reported[3–7].

Meta-analysis, a statistical approach to estimate a parameter of interest based on multiple studies, plays an essential role in medical research. One consequence of research underreporting is that it influences the sample of studies that is available for a meta-analysis[8,9]. This causes a bias, unless the process of study selection is modeled correctly[10]. Such modeling requires strong assumptions about the nature of the publication bias, especially when the size of a meta-analysis is not very large and when robust techniques cannot be used[11–13]. As a result, when publication bias occurs, the validity of the meta-analysis is uncertain.

It is well-known that smaller studies are often characterized by larger effects in published meta-analyses[14–16]. Publication bias is one of the possible explanations of this phenomenon[17]. Although a meta-analysis is typically preceded by an investigation of the presence of publication bias, the standard detection methods are characterized by a low power[11,18–22]. Therefore, the sample of included studies may be unrepresentative of the population of all conducted studies even when publication bias has not been detected. In this study, we investigated whether statistically significant outcomes that showed a positive effect of the treatment (in the case of clinical trials) and plausible statistically significant outcomes (in the case of observational studies and interventional studies) had a greater probability of being included in recent meta-analyses than other outcomes. We considered all meta-analyses of aggregate data that included at least 30 effect sizes from individual studies and were published between 2008 and 2012 in four major general medical journals: BMJ, JAMA, Lancet, and PLOS Medicine. We applied a Bayesian approach, which allows estimation of a parametric function that describes the selection process[23].

## Methods

For meta-analyses of clinical trials, we a priori decided to estimate the ratio of the probability of including statistically significant results favoring the treatment to the probability of including other results. For other meta-analyses, we a priori decided to estimate the ratio of the probability of including plausible statistically significant results to the probability of including other results. The definition of plausibility was straightforward and a priori chosen. For meta-analyses of an association between a risk factor and an undesired outcome (disease, mortality, etc.), results showing a positive association were regarded as plausible. For meta-analyses of an association between the absence of a risk factor and an undesired outcome, results showing a negative association were regarded as plausible. In meta-analyses of associations between alcohol consumption and cardiovascular parameters, we estimated the ratio of the probability of including statistically significant results to the probability of including not statistically significant results because both a positive and negative association is biologically plausible[24,25]. A two-sided significance level of 0.05 was assumed. Further in the article, we refer to the ratio of probabilities as the relative risk (RR).

### Identification of meta-analyses

We used PubMed to identify meta-analyses published between 2008 and 2012 in four general medical journals (BMJ, JAMA, Lancet, and PLOS Medicine). The term ‘meta-analysis’ was required to appear in the title or the abstract. Meta-analyses that combined at least 30 estimates from individual studies were considered. Only large meta-analyses were included because a substantial sample size is required to distinguish between the effects of heterogeneity and publication bias[11,26]. We considered only meta-analyses of aggregate data because a selection model based on p-values seemed to be inappropriate to study the complicated process of study selection in individual participant data meta-analyses. 

### Statistical model

For each identified meta-analysis, we applied a hierarchical selection model[23,27]. In this approach, a weight function is incorporated in a meta-analysis to model the probability that an individual study is included. Similarly as in the case of a standard random effects meta-analysis, the conditional distribution of the observed study effects Y_i_ (i=1, …, N), given the true study effect α_i_ and the within-study variance σ_i_^2^, is assumed to be normal: f(y_i_|α_i_,σ_i_^2^)~N(α_i_,σ_i_^2^). The true study effect comes from a normal distribution: N(µ,τ^2^), were µ is the mean effect size and τ^2^ is the between-study variance. When a selection process is present some studies may fail to enter the meta-analysis sample. To model the process of study selection, the probability that any specific study enters the sample is assumed to be multiplied by a nonnegative weight function. In this case, the observed study effects X_j_ that enter the meta-analysis sample (j=1, …, n, n≤N) have a weighted density: f^w^(x_j_|α_j_,σ_j_^2^)=w(x_j_)f(x_j_|α_j_,σ_j_^2^)/∫w(x)f(x|α_j_,σ_j_^2^)dx, where w(x) is a weight function. We applied a step weight function that took two values: ϒ_1_ for statistically significant results favoring the treatment (in the case of clinical trials) or plausible statistically significant results (in the case of other studies) and ϒ_2_ for other results, so that the RR equaled ϒ_1_/ϒ_2_

Maximum likelihood estimation is one possible approach to fit the model described above[27,28]. We used Bayesian inference because it produces valid results when the sample size is small[29], allows a straightforward interval estimation, and examination of the sensitivity of the findings to the distribution of the random effects. Similarly to Silliman[23], we used diffuse uniform priors U(0,1) for the parameters of the weight function. We declared a diffuse prior N(0,1000) for the mean effect size and, following a recommendation of Gelman[30], a uniform prior for the between-study standard deviation. We used Gibbs sampling [31] to obtain samples from the posterior distribution of ϒ_1_/ϒ_2_. We applied the algorithm described by Silliman, who considered a general class of hierarchical selection models[23]. In our specific case, the full conditional distributions needed by the Gibbs sampler were:

$\alpha_{j}|x,\sigma ,\alpha_{k\neq j},\mu ,\tau^{2},ϒ\propto c_{\omega ,\alpha_{j},\sigma_{j},ϒ}{}^{-1}N\left(x_{j}\frac{\tau^{2}}{\tau^{2}+\sigma_{{}_{j}}^{2}}+\mu \frac{\sigma_{{}_{j}}^{2}}{\tau^{2}+\sigma_{{}_{j}}^{2}},\;\frac{\tau^{2}\sigma_{{}_{j}}^{2}}{\tau^{2}+\sigma_{{}_{j}}^{2}}\right),\;j=1,...,n$

$$\mu |x,\sigma ,\alpha ,\tau^{2},ϒ\propto N\left(\sum_{j}^{n}\alpha_{j}\frac{1000}{\tau^{2}+1000n},\;\frac{1000\tau^{2}}{\tau^{2}+1000n}\right)$$

$$\tau^{2}|x,\sigma ,\alpha ,\mu ,ϒ\propto IG(\frac{1}{2}(n-1),\;\frac{1}{2}\sum_{j=1}^{n}{\left(\alpha_{j}-\mu \right)}^{2})$$

${ϒ}_{1}|x,\sigma ,\alpha ,\mu ,\tau^{2},{ϒ}_{2}\propto \frac{{ϒ}_{1}{}^{s}}{\prod_{j=1}^{n}c_{\omega ,\alpha_{j},\sigma_{j},ϒ}}$, and

$${ϒ}_{2}|x,\sigma ,\alpha ,\mu ,\tau^{2},{ϒ}_{1}\propto \frac{{ϒ}_{2}{}^{n-s}}{\prod_{j=1}^{n}c_{\omega ,\alpha_{j},\sigma_{j},ϒ}}$$

where$c_{\omega ,\alpha_{j},\sigma_{j},ϒ}=\int w(x)f(x|\alpha_{j},\sigma_{j}{}^{2})dx$, **x**= (x_1_, ..., x_n_), **σ**= (σ_1_, ..., σ_n_), **α**= (α_1_, ..., α_n_), **ϒ**=(ϒ_1_,ϒ_2_), and s is the number of statistically significant results favoring the treatment (in the case of meta-analyses of clinical trials) or plausible statistically significant results (in the case of meta-analysis of other studies). A burn-in of 10000 iterations was sufficient to achieve convergence for all meta-analyses. The estimates were based on the subsequent 50000 iterations.

For point estimation, we used the median of the posterior distribution of RR. For interval estimation, we used the 95% equal-tail credible interval (CI). When the posterior probability that the RR exceeded 1 was greater than 0.95, we concluded that there was strong evidence that the RR was greater than 1. An R program that was used to fit the models can be found in Appendix S1.

### Quality of the statistical model

In order to evaluate whether the statistical model was suitable for the objectives of the study, we performed simulations. The settings were based on the characteristics of the meta-analyses of clinical trials included in the study (see Appendix S2). The posterior distribution provided reliable information about the size of the RR (Table 1). Although the estimate of the RR based on the median of the posterior distribution was characterized by a substantial variability and was biased in some scenarios, it gave a correct idea about the existence of a publication bias. The model tended to underestimate the RR when the mean effect size was small and overestimate the RR when the mean effect size was large. However, it was able to distinguish the RR from the mean effect size well for all simulation settings, as indicated by the quality of the interval estimates of the RR. For almost all scenarios, the lower bound of the 95% equal-tail credible interval was smaller than the assumed true value of the RR in at least 95% of the simulations. Although the upper bound was sometimes too small when the mean effect size was small, it was greater than the true RR in more than 95% of the simulations for most of the scenarios (Table 1). The performance of the model did not depend on the size of the between-study heterogeneity. The model was robust to the presence of small study effects (i.e., larger true effects in smaller studies, Table 1). 

**Table 1:** Model performance: quality of estimation.

**RR**	**SSE**	**I^2^**	**µ**	**Bias**	**ME**	**MSE**	**LB**	**UB**	**Total**
1	No	0.36	0.0	-0.07	0.36	0.24	0.999	1.000	0.999
1	No	0.36	0.2	-0.03	0.33	0.21	0.995	0.995	0.990
1	No	0.36	0.8	0.09	0.32	0.22	0.981	0.989	0.970
1	No	0.36	1.4	0.21	0.38	0.38	0.965	0.994	0.959
1	No	0.67	0.0	-0.02	0.35	0.27	0.996	0.996	0.992
1	No	0.67	0.2	0.01	0.33	0.22	0.994	0.994	0.988
1	No	0.67	0.8	0.10	0.32	0.24	0.984	0.993	0.977
1	No	0.67	1.4	0.25	0.42	0.45	0.966	0.994	0.960
4	No	0.36	0.0	-1.45	1.98	5.21	0.997	0.853	0.850
4	No	0.36	0.2	-1.07	1.71	3.96	0.993	0.855	0.848
4	No	0.36	0.8	0.26	1.86	8.32	0.982	0.952	0.934
4	No	0.36	1.4	0.93	2.74	28.9	0.978	0.967	0.945
4	No	0.67	0.0	-1.06	1.93	5.56	0.996	0.876	0.872
4	No	0.67	0.2	-0.89	1.71	4.13	0.997	0.903	0.900
4	No	0.67	0.8	0.65	2.15	13.4	0.971	0.968	0.939
4	No	0.67	1.4	1.41	2.97	37.9	0.978	0.977	0.955
10	No	0.36	0.0	-3.38	4.51	26.5	0.996	0.831	0.827
10	No	0.36	0.2	-2.52	4.14	24.5	0.995	0.871	0.866
10	No	0.36	0.8	2.63	6.99	209	0.979	0.954	0.933
10	No	0.36	1.4	0.52	6.33	75.0	1.000	0.962	0.962
10	No	0.67	0.0	-2.90	4.52	28.6	0.997	0.866	0.863
10	No	0.67	0.2	-1.79	4.42	30.5	0.994	0.902	0.896
10	No	0.67	0.8	4.52	8.35	269	0.973	0.970	0.943
10	No	0.67	1.4	2.72	7.64	153	0.995	0.968	0.963
1	Yes	0.36	0.0	-0.03	0.42	0.38	0.998	0.998	0.996
1	Yes	0.36	0.2	0.07	0.40	0.41	0.987	0.992	0.979
1	Yes	0.36	0.8	0.30	0.42	0.41	0.950	0.995	0.945
1	Yes	0.36	1.4	0.46	0.53	0.64	0.931	0.998	0.929
1	Yes	0.67	0.0	0.05	0.41	0.37	0.996	0.997	0.993
1	Yes	0.67	0.2	0.06	0.38	0.32	0.992	0.992	0.984
1	Yes	0.67	0.8	0.25	0.40	0.39	0.965	0.999	0.964
1	Yes	0.67	1.4	0.46	0.54	0.74	0.942	0.997	0.939
4	Yes	0.36	0.0	-1.17	2.10	6.15	0.997	0.878	0.875
4	Yes	0.36	0.2	-0.41	1.83	5.79	0.989	0.922	0.911
4	Yes	0.36	0.8	1.59	2.50	19.3	0.947	0.982	0.929
4	Yes	0.36	1.4	2.39	3.34	41.1	0.968	0.991	0.959
4	Yes	0.67	0.0	-0.79	2.00	6.28	0.995	0.909	0.904
4	Yes	0.67	0.2	-0.51	1.74	5.06	0.992	0.935	0.927
4	Yes	0.67	0.8	1.65	2.64	20.4	0.957	0.981	0.938
4	Yes	0.67	1.4	2.57	3.57	45.9	0.956	0.987	0.943
10	Yes	0.36	0.0	-2.00	4.72	37.3	0.992	0.888	0.880
10	Yes	0.36	0.2	-0.69	4.22	30.9	0.986	0.905	0.891
10	Yes	0.36	0.8	6.56	9.00	350	0.959	0.991	0.950
10	Yes	0.36	1.4	3.46	7.23	119	1.000	0.987	0.987
10	Yes	0.67	0.0	-1.17	4.35	30.0	0.996	0.918	0.914
10	Yes	0.67	0.2	-0.41	4.39	35.3	0.988	0.937	0.925
10	Yes	0.67	0.8	6.44	9.35	379	0.956	0.983	0.939
10	Yes	0.67	1.4	5.66	9.38	214	0.995	0.989	0.984

RR: relative risk, the ratio of the probability of including statistically significant outcomes favoring the treatment to the probability of including other outcomes (for RR=1, all results had the same probability of being included), SSE: small study effect, I^2^: proportion of the total variability due to heterogeneity, µ: mean effect size, Bias: average difference between the median of the posterior distribution of the RR and the true RR, ME: mean error, MSE: mean squared error, LB: proportion of the lower bounds of the 95% equal-tail credible intervals lower than the true RR, UB: proportion of the upper bounds of the 95% equal-tail credible intervals greater than the true RR, Total: proportion of the 95% equal-tail credible intervals including the true RR.

Additionally, we compared the ability of our model to detect a selection process based on the statistical significance with publication bias methods widely used in medical research: the Egger’s test[32], correlation test of Begg and Mazumdar[33], and the trim and fill method[34]. When statistically significant outcomes had a higher probability of being included, the Bayesian selection model showed much higher detection rates compared to the standard methods (Table 2). This difference was especially apparent when small study effects were absent. Furthermore, in contrast to the standard methods, the Bayesian selection model was characterized by low false positive rates, even in the presence of small-study effects (Table 2).

**Table 2:** Model performance: type 1 error and power.

**RR**	**SSE**	**I^2^**	**µ**	**Current model**	**Egger**	**Rank correlation**	**Trim and Fill**
1	No	0.36	0.0	0.008	0.048	0.019	0.027
1	No	0.36	0.2	0.012	0.059	0.027	0.032
1	No	0.36	0.8	0.039	0.078	0.032	0.066
1	No	0.36	1.4	0.058	0.084	0.044	0.041
1	No	0.67	0.0	0.009	0.047	0.019	0.012
1	No	0.67	0.2	0.009	0.054	0.023	0.031
1	No	0.67	0.8	0.030	0.085	0.026	0.045
1	No	0.67	1.4	0.056	0.093	0.038	0.046
4	No	0.36	0.0	0.308	0.049	0.017	0.043
4	No	0.36	0.2	0.560	0.068	0.024	0.055
4	No	0.36	0.8	0.829	0.573	0.446	0.231
4	No	0.36	1.4	0.711	0.396	0.423	0.277
4	No	0.67	0.0	0.415	0.030	0.004	0.016
4	No	0.67	0.2	0.567	0.051	0.006	0.028
4	No	0.67	0.8	0.794	0.471	0.273	0.170
4	No	0.67	1.4	0.730	0.370	0.288	0.225
10	No	0.36	0.0	0.922	0.084	0.029	0.073
10	No	0.36	0.2	0.979	0.391	0.274	0.111
10	No	0.36	0.8	0.989	0.816	0.783	0.470
10	No	0.36	1.4	0.953	0.588	0.608	0.527
10	No	0.67	0.0	0.939	0.061	0.020	0.029
10	No	0.67	0.2	0.971	0.309	0.177	0.061
10	No	0.67	0.8	0.986	0.733	0.637	0.354
10	No	0.67	1.4	0.958	0.492	0.512	0.401
1	Yes	0.36	0.0	0.007	0.190	0.089	0.094
1	Yes	0.36	0.2	0.020	0.233	0.093	0.098
1	Yes	0.36	0.8	0.098	0.241	0.131	0.154
1	Yes	0.36	1.4	0.131	0.240	0.126	0.140
1	Yes	0.67	0.0	0.009	0.142	0.045	0.070
1	Yes	0.67	0.2	0.020	0.170	0.053	0.069
1	Yes	0.67	0.8	0.060	0.193	0.067	0.112
1	Yes	0.67	1.4	0.105	0.216	0.078	0.108
4	Yes	0.36	0.0	0.304	0.241	0.115	0.165
4	Yes	0.36	0.2	0.592	0.271	0.085	0.152
4	Yes	0.36	0.8	0.926	0.840	0.636	0.432
4	Yes	0.36	1.4	0.894	0.680	0.615	0.467
4	Yes	0.67	0.0	0.424	0.092	0.028	0.071
4	Yes	0.67	0.2	0.603	0.141	0.018	0.087
4	Yes	0.67	0.8	0.890	0.680	0.439	0.261
4	Yes	0.67	1.4	0.846	0.550	0.424	0.345
10	Yes	0.36	0.0	0.885	0.382	0.163	0.252
10	Yes	0.36	0.2	0.986	0.580	0.321	0.247
10	Yes	0.36	0.8	0.999	0.950	0.923	0.641
10	Yes	0.36	1.4	0.988	0.807	0.784	0.689
10	Yes	0.67	0.0	0.956	0.159	0.045	0.088
10	Yes	0.67	0.2	0.988	0.400	0.227	0.134
10	Yes	0.67	0.8	0.994	0.868	0.791	0.492
10	Yes	0.67	1.4	0.983	0.715	0.636	0.538

RR: relative risk, the ratio of the probability of including statistically significant outcomes favoring the treatment to the probability of including other outcomes (for RR=1, all results had the same probability of being included), SSE: small study effect, I^2^: proportion of the total variability due to heterogeneity, µ: mean effect size. Proportion of meta-analyses, in which publication bias was identified, is presented. For the Bayesian selection model, publication bias was indicated when the posterior probability that the RR was larger than 1 exceeded 95%. For the Egger’s test and the rank correlation test, one-sided procedures were used with a 0.05 significance level. For the trim and fill method, publication bias was indicated when the number of missing studies estimated by the R estimator in the first step of the algorithm was greater than 3[34].

### Sensitivity analysis

In order to investigate the robustness of the findings, two alternative models were considered. Because the conclusions drawn from a hierarchical model may be sensitive to the choice of the prior for the variance of the random effects[35], in the first model, we replaced the uniform prior for τ with a 1/τ^2^ prior for τ^2^ (for an R program: see Appendix S1). In the second model, the assumption of a normal distribution of α_i_ was relaxed by allowing it to follow a t-distribution. A prior U(2,100) was declared for the number of degrees of freedom. A Winbugs program that was used to fit this model can be found in Appendix S3.

## Results

### Identification of meta-analyses

**Figure 1:**
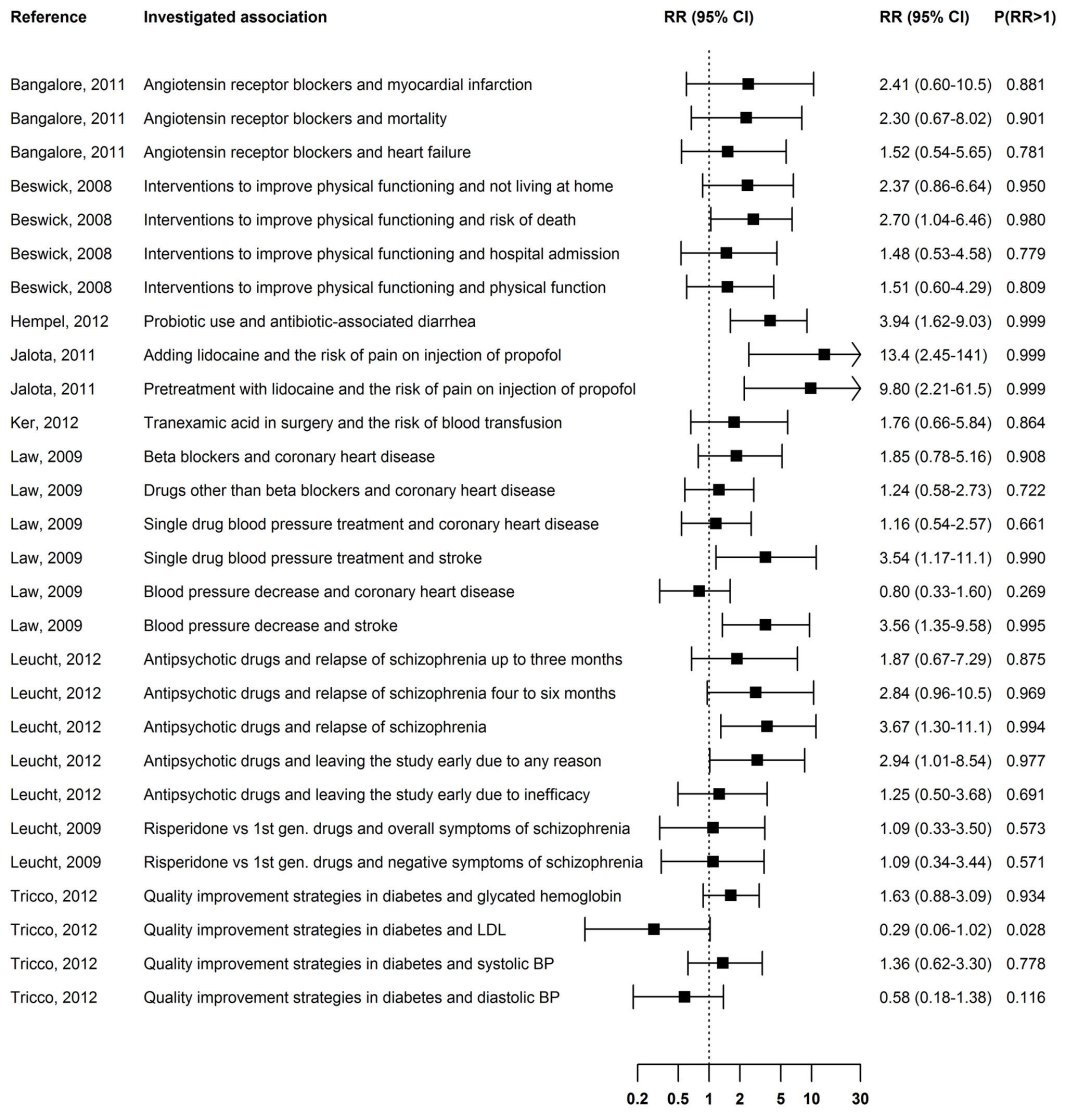
Publication bias in meta-analyses of clinical trials. The RR is the ratio of the probability of including statistically significant results favoring the treatment to the probability of including other results. The median of the posterior distribution was used for point estimation. The interval estimate is the 95% equal-tail credible interval. P(RR>1) is the posterior probability that statistically significant results favoring the treatment had a higher chance of being included in the meta-analysis than other results.

**Figure 2:**
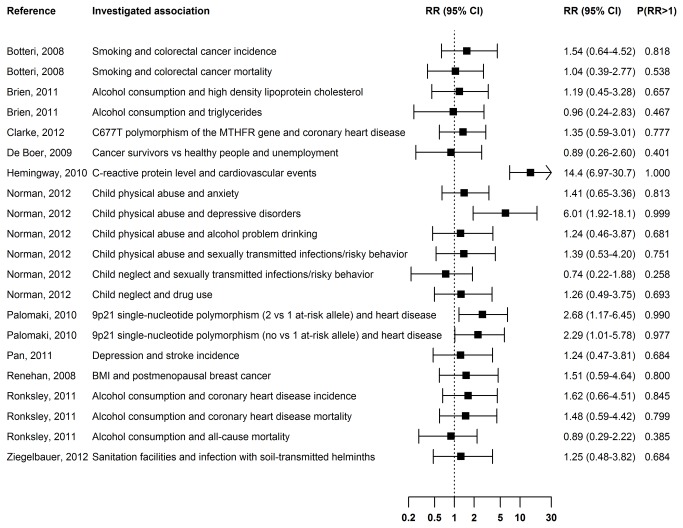
Publication bias in meta-analyses of studies other than clinical trials. The RR is the ratio of the probability of including plausible statistically significant results to the probability of including other results. The median of the posterior distribution was used for point estimation. The interval estimate is the 95% equal-tail credible interval. P(RR>1) is the posterior probability that plausible statistically significant results had a higher chance of being included in the meta-analysis than other results.

Out of 406 articles that were identified in the initial search, 88 articles did not report meta-analyses of an association. We excluded 280 articles because they did not describe a meta-analysis including at least 30 effect sizes. Further, 14 articles did not report any results from a meta-analysis of aggregate data. Finally, four articles were excluded because they did not report the effect sizes from the individual studies and the corresponding author did not respond to a request to provide them. Twenty reports including 49 meta-analyses were used in this study (Figure 1 and Figure 2, references: Appendix S4, raw data available at www.plosone.org).

### RR in meta-analyses of clinical trials

**Figure 3:**
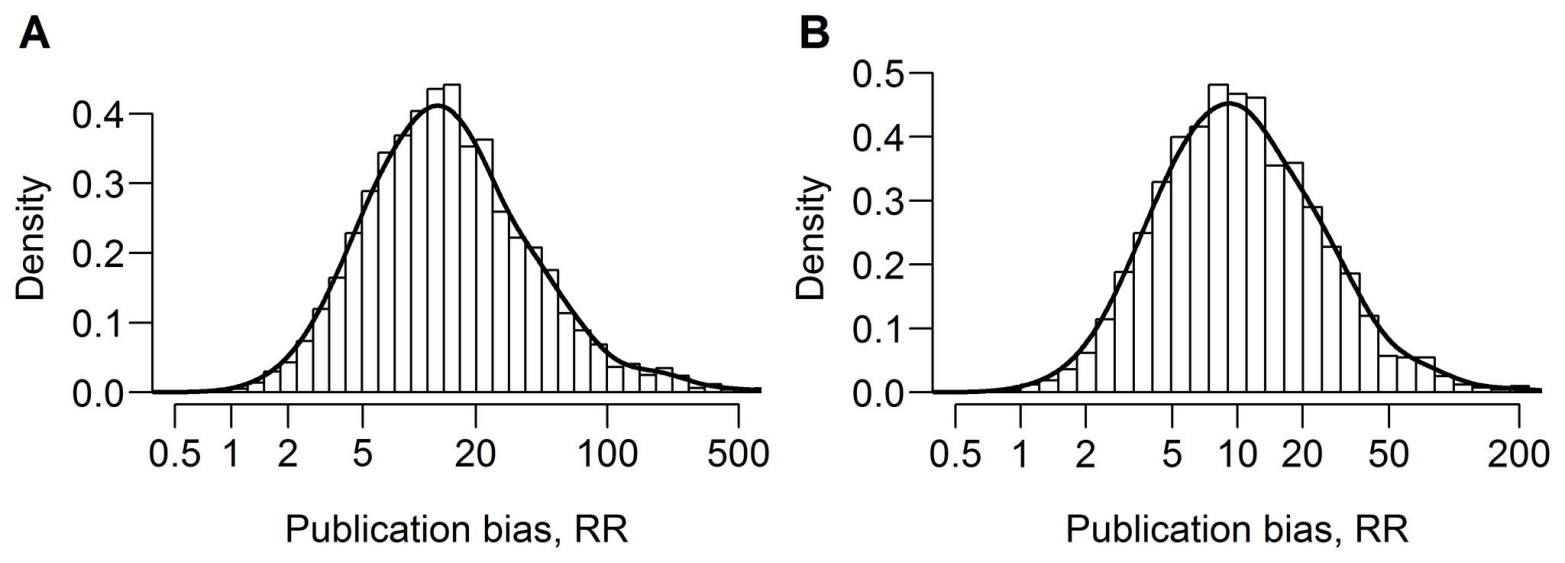
The posterior distribution of the RR in the meta-analyses of the associations between A) adding lidocaine and the risk of pain on injection of propofol, and B) pretreatment with lidocaine and the risk of pain on injection of propofol. The posterior distributions describe the knowledge about the RR. The higher the value of the density function, the more likely a given value of RR is in light of the prior knowledge (no prior knowledge was assumed) and the data from the meta-analysis. For both meta-analyses, there was much certainty that the RR was greater than 1, indicating that statistically significant results favoring the treatment had a greater probability of being included in the meta-analysis than other results.

We estimated the ratio of the probability of including statistically significant results favoring the treatment to the probability of including other results in 28 large meta-analyses of clinical trials that were described in nine articles published in BMJ, JAMA, Lancet, or PLOS Medicine from 2008 to 2012 (Figure 1). In 25 out of the 28 meta-analyses, the estimate of the RR was greater than 1. In 10 meta-analyses, there was strong evidence that statistically significant results favoring the treatment had a higher probability of being included than other outcomes (Figure 1). Trials that demonstrated the efficacy of adding lidocaine on prevention of pain on injection of propofol were estimated to be between 2.45 and 141 times more likely to be included in the meta-analysis than other trials (Figure 3A). Studies favoring pretreatment with lidocaine were estimated to have a between 2.21 and 61.5-fold higher probability of being included in the meta-analysis than other studies (Figure 3B). Changing the prior distribution for the between-study variance and the distribution of the true study effects had little effect on the estimates (Appendix S5).

### RR in other meta-analyses

**Figure 4:**
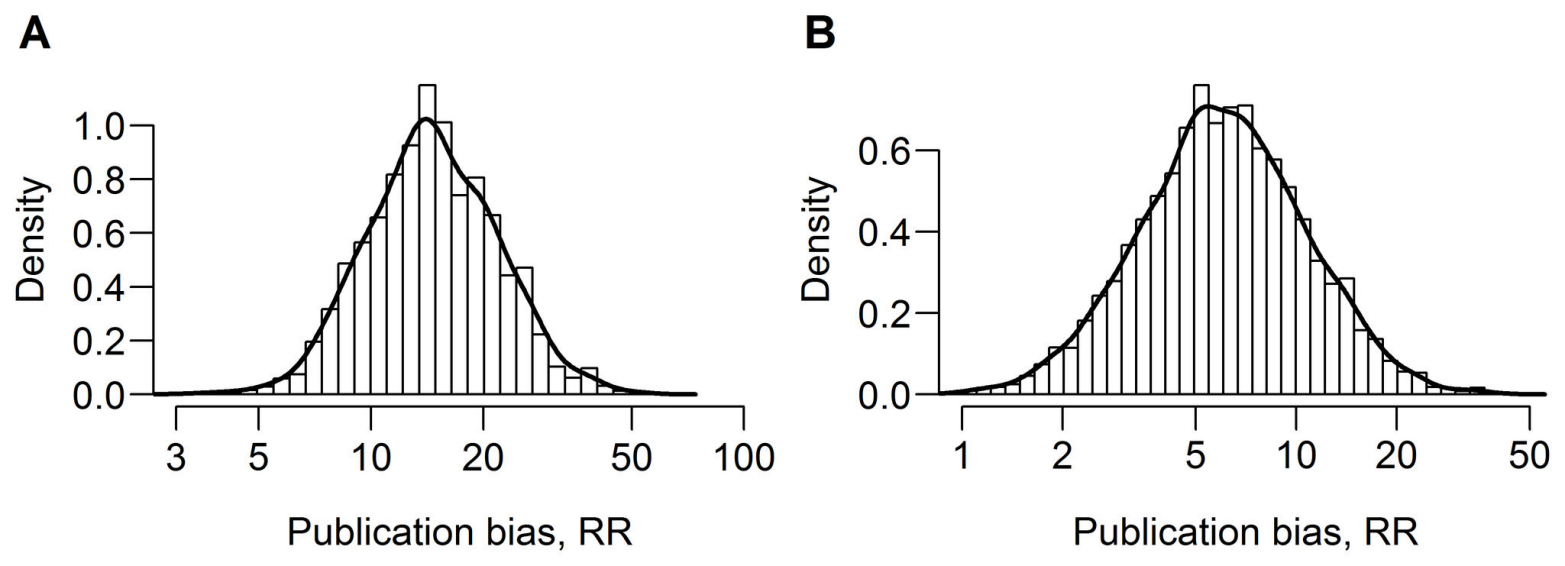
The posterior distribution of the RR in the meta-analyses of the associations between A) c−reactive protein level and cardiovascular events and B) child physical abuse and depressive disorders. The posterior distributions describe the knowledge about the RR. The higher the value of the density function, the more likely a given value of RR is in light of the prior knowledge (no prior knowledge was assumed) and the data from the meta-analysis. For both meta-analyses, there was much certainty that the RR was greater than 1, indicating that plausible statistically significant results had a greater probability of being included in the meta-analysis than other results.

We identified 10 articles describing 19 meta-analyses of observational studies and one article describing two meta-analyses of interventional studies that were published in BMJ, JAMA, Lancet, or PLOS Medicine between 2008 and 2012. In four meta-analyses, there was strong evidence that plausible statistically significant results had a higher probability of being included than other outcomes (Figure 2). Studies that showed a statistically significant positive association between the C−reactive protein level and cardiovascular events were estimated to have a between 6.97 and 30.7-fold higher probability of being included in the meta-analysis than studies showing other outcomes (Figure 4A). Statistically significant results showing a positive association were estimated to be between 1.92 and 18.1 times more likely to be included in the meta-analysis on the association between child physical abuse and depressive disorders (Figure 4B). The results were robust to the choice of the prior distribution for the between-study variance and the assumption about the distribution of the true study effects (Appendix S6).

## Discussion

Clinical trials showing statistically significant results favoring the treatment and observational studies showing plausible statistically significant outcomes often had a higher probability of being included in the recent meta-analyses than studies showing other results. The magnitude of the publication bias differed greatly between the meta-analyses and was very large in some cases. For example, for a meta-analysis of the association between the C−reactive protein level and cardiovascular events, statistically significant outcomes showing a positive association were estimated to be between 6.97 and 30.7 times more likely to be included in the analyzed sample than other results.

The effect of the higher probability of including for statistically significant outcomes on the combined estimates is unknown due to a lack of information about the exact nature of the bias. However, it is clear that the fundamental assumption of a lack of systematic bias in the process of study selection was strongly violated. Consequently, the validity of a substantial proportion of the recent meta-analyses published in major general medical journals is uncertain due to the presence of a publication bias. Only in 3 [36–38] out of the 14 meta-analyses, in which we found evidence that statistically significant outcomes had a higher probability to be included, the presence of a publication bias was acknowledged in the article.

The study demonstrates an application of an attractive alternative to the standard publication bias detection methods for studying a selection process based on the statistical significance in large meta-analyses. Widely used publication bias methods such as the trim and fill method[34,39], Egger’s test[32], rank correlation test[33], and their modifications [18,19,40–42] are based on funnel plot asymmetry. These approaches have two major disadvantages. First, funnel plot asymmetry may be caused by processes other than publication bias, such as between-study variability or small study effects (i.e., larger true effects in smaller studies)[17,32]. As a result, these methods may incorrectly suggest that a publication bias is present[13,22,43,44]. Second, some selection processes introduce little asymmetry to the funnel plot. As a result, widely used publication bias tests often have a low power[18,20,22]. In contrast to these methods, selection models do not rely on the funnel plot but incorporate a model for publication bias in the random effects meta-analysis. While standard approaches investigate the association between effect sizes and some measure of precision to draw conclusions about publication bias, selection models allow to directly estimate parameters that describe the selection process.

Several alternatives to the methods based on the funnel plot have been suggested. Iyengar and Greenhouse introduced selection models in meta-analysis[45]. Hedges proposed a class of selection models that incorporated between-study variance[27]. The advantage of these two frequentist methods compared to the class of Bayesian hierarchical selection models described by Silliman [23] is their computational simplicity. We chose the Bayesian approach because it produces valid conclusions when the sample size is small[29], allows a straightforward interval estimation and examination of the sensitivity of the findings to the distribution of the random effects. When a selection process based on the p-values is a point of interest but the weight function is difficult to specify a priori, non-parametric selection models can be used[46,47]. In this study, a parametric model was applied because the aim was to estimate a specific parametric function. Ioannidis and Trikalinos introduced a method based on a comparison of the number of expected and observed statistically significant results in a meta-analysis[48]. A major advantage of this approach is that it does not require a large sample size. An advantage of selection models compared to the method of Ioannidis and Trikalinos is that they allow to take between-study variance into account.

Different selection mechanisms have been considered. Dear and Begg developed a selection model based on the assumption that the probability of publishing can be described with a step function with discontinuities at alternate observed p-values[46]. Rufibach described a method that imposed a monotonicity constrained on this function[47]. The trim and fill method handles publication bias defined as the absence of studies with most extreme negative estimates[34,39]. The model of Copas and Shi assumes that the selection probability, given the size of the study, is an increasing function of the observed study effect[49]. Ioannidis and Trikalinos developed a test to investigate an excess of statistically significant findings[48]. We investigated whether statistically significant results favoring the treatment had a higher probability of being included in the meta-analyses of clinical trials. We focused on this selection process because its existence in the medical literature is well-documented by empirical studies following research from inception or submission to a regulatory authority[4,6]. In the case of other meta-analyses, we estimated the ratio of the probability of including biologically plausible statistically significant results to the probability of including other results. As demonstrated by the simulation study, our model performed well in detecting a selection process based on the statistical significance and direction of the effect. However, the power of the model to detect publication bias may be lower when a selection mechanism of a different nature occurs. 

The main limitation of the study is that we focused on the largest meta-analyses. Possibly, the size of the association between the statistical significance of the results and the probability of including is different for small and medium meta-analyses than for the largest meta-analyses that we considered.

When publication bias is detected, an analyst can attempt to account for it[23,28,39,47,49–51]. Although the methods to conduct meta-analysis in the presence of a publication bias provide a powerful sensitivity analysis tool, their validity depends on the correctness of strong and unverifiable assumptions[12]. In light of the studies on publication bias in medicine, including the one presented here, it is clear that the quality of evidence from medical research greatly benefits from policies that aim to reduce underreporting. Several measures that regulate clinical trials have been recently taken. Since 2005, the International Committee of Medical Journal Editors requires a prospective public registration of clinical trials as a condition for publication. Since 2007, the U.S. Food and Drug Administration has also required the registration of trial results. Similar initiatives are needed for observational studies in order to make a clear distinction between a predefined hypothesis testing and exploratory analysis[52]. A prospective registration of all study protocols including a detailed description of the data analysis, a requirement of consistency between the protocol and the study report, and an obligatory disclosure of the results are recommended to further improve the quality of medical literature.

## Supporting Information

Appendix S1 **R code to fit the main model and the model with a modified prior for the between-study variance.**
(TXT)

Appendix S2 **Simulation details.**
(PDF)

Appendix S3 **Winbugs code for the model with t-distributed study-specific means.**
(PDF)

Appendix S4 **References of meta-analyses included in the study.**
(PDF)

Appendix S5 **Sensitivity analysis for meta-analyses of clinical trials.**
(PDF)

Appendix S6 **Sensitivity analysis for meta-analyses of observational and interventional studies.**
(PDF)

Appendix S7 **Raw data.**
(ZIP)
