# Correction: Publication Bias in Recent Meta-Analyses

**DOI:** 10.1371/annotation/51ecf224-b045-4424-8beb-de155769d429

**Published:** 2013-12-20

**Authors:** Michal Kicinski

There were errors in multiple equations in the article. On the left side of all "conditional distribution" equations (located in the second paragraph of the "Statistical model" section of the Methods), the "vector" terms "**x**,""**α**,"**σ**," and "**ϒ**" are not correctly bolded. In the first listed of these equations, the power "-1" should refer to the term "c," not the term "**ϒ**" as is currently indicated. In the first-listed of these equations, there is an unnecessary space between the right bracket and the last term "2". In the fourth-listed of these equations, the power "s" should refer to the term "**ϒ**," not the term "1" as currently indicated. In the fifth-listed of these equations, the power "n-s" should refer to the term "**ϒ**," not the term "2" as currently indicated. In the sentence introducing these equations, the word "where" and the term "c" are incorrectly joined, and spaces were incorrectly inserted before the second, third and fourth pairs of brackets. 

