# Correction: Publication Bias in Recent Meta-Analyses

**DOI:** 10.1371/annotation/a65c0f61-eb99-42f0-828b-5a8662bce4f7

**Published:** 2014-01-13

**Authors:** Michal Kicinski

The errors in equations noted in the previous correction notice were due to typesetting errors; the publisher apologizes for these errors. In addition to the explanation in the previous notice, please see the complete, corrected paragraph and equations here: http://www.plosone.org/corrections/pone.0081823.001.cn.docx

