# Supplementary material for: Publication Bias in Recent Meta-Analyses
Source: PLoS One. 2013 Nov 27;8(11):e81823. doi: 10.1371/journal.pone.0081823 (PMC3868709; doi:10.1371/journal.pone.0081823)
Supplement: Appendix S2 — Simulation details. (PDF) [file pone.0081823.s002.pdf]

APPENDIX 2: SIMULATION DETAILS

We simulated clinical trials with two arms and a binary outcome. The sample size of the individual studies was based on the sample size of clinical trials included in 22453 meta-analyses from the Cochrane Database of Systematic Reviews.[1] The number of participants came from a uniform distribution U(10,44) with a probability of 1/4, U(45, 91) with a probability of 1/4, U(92, 210) with a probability of 1/4, or U(211, 2000) with a probability of 1/4. The size of the treatment group and the control group was the same. The probability of the event in the control group was sampled from a uniform distribution U(0.3,0.7). The small study effect was modeled as an effect of the standard deviation on the study specific mean. The study specific means came from a normal distribution: $\alpha_i|\mu,SSE,\sigma_i,\tau^2 \sim N(\mu+SSEx(\sigma_i-\sigma),\tau^2)$, where σ_i is the standard deviation, and σ is the mean standard deviation of the observed study effects. The number of the events in the treatment and control group was sampled from a Binomial distribution.

The parameter values were based on the characteristics of 21 meta-analyses of clinical trials included in the study, in which the main outcome was a measure of the relative risk for event in the treatment group compared to the control group. The values considered for μ and I^2 equaled to the 25th and 75th percentiles of the random effects model estimates. For μ we additionally considered μ=0 and μ =1.4 (the largest random effects model estimate) in order to investigate the performance of the model when the mean effect was very small and very large. In the conditions without small-study effects, SSE equaled 0. In the conditions with small study effects, SSE equaled 0.3. This value was the median regression coefficient describing the association between the standard deviations and the observed study effects in the meta-analyses. The sample size equaled 39, which was the median sample size. The value of four was chosen for the RR because of the empirical evidence showing that positive results often had an approximately four times higher probability of being published than other results.[2–5] The scenarios with RR=10 simulate situations with a strong selection process. For each scenario, 1000 simulations were performed.

Reference List

1. Davey J, Turner RM, Clarke MJ, Higgins JP (2011) Characteristics of meta-analyses and their component studies in the Cochrane Database of Systematic Reviews: a cross-sectional, descriptive analysis. BMC Med Res Methodol 11: 160. 1471-2288-11-160 [pii];10.1186/1471-2288-11-160 [doi].

2. Decullier E, Lheritier V, Chapuis F (2005) Fate of biomedical research protocols and publication bias in France: retrospective cohort study. BMJ 331: 19-22. doi:10.1136/bmj.38488.385995.8F.

3. Lee K, Bacchetti P, Sim I (2008) Publication of Clinical Trials Supporting Successful New Drug Applications: A Literature Analysis. PLoS Med 5: e191. doi:10.1371/journal.pmed.0050191.

4. Song F, Parekh-Bhurke S, Hooper L, Loke Y, Ryder J, Sutton A, Hing C, Harvey I (2009) Extent of publication bias in different categories of research cohorts: a meta-analysis of empirical studies. BMC Med Res Methodol 9: 79. 10.1186/1471-2288-9-79.

5. Bardy AH (1998) Bias in reporting clinical trials. Br J Clin Pharmacol 46: 147-150.
