# Supplementary material for: Publication Bias in Recent Meta-Analyses
Source: PLoS One. 2013 Nov 27;8(11):e81823. doi: 10.1371/journal.pone.0081823 (PMC3868709; doi:10.1371/journal.pone.0081823)
Supplement: Appendix S3 — Winbugs code for the model with t-distributed study-specific means. (PDF) [file pone.0081823.s003.pdf]

APPENDIX 3: WINBUGS CODE FOR THE MODEL WITH T-DISTRIBUTED STUDY-SPECIFIC MEANS

Parameters:

x: observed effect sizes
s: the corresponding standard deviations
N: number of observed effect sizes
mu: mean effect size
alpha: study-specific means
v1, v2: parameters of the weight function
bs_sd: the square root of the between-study variance
df: number of degrees of freedom of the distribution of the study-specific means

The model for RR=P(including statistically significant positive outcomes)/P(including other outcomes):

```
 model
{
C <- 10000 ## this just has to be large enough to ensure all ph[i]'s > 0
for(i in 1:N)
	{
	xs[i]<-step(x[i]/s[i]-1.96)
	q_std[i]<-(1.96*s[i]-alpha[i])/s[i]
	CDFq[i]<-phi(q_std[i])
	minl[i]<- -log(xs[i]*v1+(1-xs[i])*v2)+0.5*pow((x[i]-alpha[i])/s[i],2)+log(v1*(1-
CDFq[i])+v2*CDFq[i]) ## -log likelihood

	## the zero's trick, see: Winbugs help
	zeros[i]<-0
	zeros[i]~dpois(ph[i])
	ph[i]<-minl[i]+C

	alpha[i]~dt(mu,bs_prec,df)
	}
RR<-v1/v2

## priors
mu~dnorm(0,0.000001)
bs_sd~dunif(0,10)
bs_var<-bs_sd*bs_sd
bs_prec<-1/bs_var
v1~dunif(0,1)
v2~dunif(0,1)
df~dunif(2,100)
}
```

The model for RR=P(including statistically significant negative outcomes) / P(including other outcomes):

```
model
{
C <- 10000 ## this just has to be large enough to ensure all ph[i]'s > 0
for(i in 1:N)
	{
	xns[i]<-1-step(x[i]/s[i]+1.96)
	q_std[i]<-(-1.96*s[i]-alpha[i])/s[i]
	CDFq[i]<-phi(q_std[i])
	minl[i]<- -log(xns[i]*v1+(1-xns[i])*v2)+0.5*pow((x[i]-alpha[i])/s[i],2)+log(v1*CDFq[i]+v2*(1-
CDFq[i])) ## -log likelihood

	## the zero's trick, see: Winbugs help
	zeros[i] <- 0
	zeros[i] ~ dpois(ph[i])
	ph[i] <- minl[i] + C

	alpha[i]~dt(mu,bs_prec,df)
	}
RR<-v1/v2

## priors
mu~dnorm(0,0.000001)
bs_sd~dunif(0,10)
bs_var<-bs_sd*bs_sd
bs_prec<-1/bs_var
v1~dunif(0,1)
v2~dunif(0,1)
df~dunif(2,100)
}
```

The model for RR=P(including statistically significant outcomes) / P(including other outcomes):

```
model
{
C <- 10000 ## this just has to be large enough to ensure all ph[i]'s > 0
for(i in 1:N)
	{
	xs[i]<-step(abs(x[i]/s[i])-1.96)
	CDF[i]<-phi((-1.96*s[i]-alpha[i])/s[i])+1-phi((1.96*s[i]-alpha[i])/s[i])
	minl[i]<- -log(xs[i]*v1+(1-xs[i])*v2)+0.5*pow((x[i]-alpha[i])/s[i],2)+log(v1*CDF[i]+v2*(1-
CDF[i])) ## -log likelihood

	## the zero's trick, see: Winbugs help
	zeros[i]<-0
	zeros[i]~dpois(ph[i])
	ph[i]<-minl[i]+C
```

```
		alpha[i]~dt(mu,bs_prec,df)
		}
RR<-v1/v2

## priors
mu~dnorm(0,0.000001)
bs_sd~dunif(0,10)
bs_var<-bs_sd*bs_sd
bs_prec<-1/bs_var
v1~dunif(0,1)
v2~dunif(0,1)
df~dunif(2,100)
}
```
