# Supplementary material for: Publication Bias in Recent Meta-Analyses
Source: PLoS One. 2013 Nov 27;8(11):e81823. doi: 10.1371/journal.pone.0081823 (PMC3868709; doi:10.1371/journal.pone.0081823)
Supplement: Appendix S4 — References of meta-analyses included in the study. (PDF) [file pone.0081823.s004.pdf]

APPENDIX 4: REFERENCES OF META-ANALYSES INCLUDED IN THE STUDY

Meta-analyses of clinical trials:

Bangalore S, Kumar S, Wetterslev J, Messerli F. Angiotensin receptor blockers and risk of myocardial infarction: meta-analyses and trial sequential analyses of 147 020 patients from randomised trials. *BMJ* 2011;342:d2234.

Beswick AD, Rees K, Dieppe P et al. Complex interventions to improve physical function and maintain independent living in elderly people: a systematic review and meta-analysis. *The Lancet* 2008;371(9614):725-735.

Hempel S, Newberry S, Maher A et al. Probiotics for the prevention and treatment of antibiotic-associated diarrhea: A systematic review and meta-analysis. *JAMA* 2012;307(18):1959-1969.

Jalota L, Kalira V, George L et al. Prevention of pain on injection of propofol: systematic review and meta-analysis. *BMJ* 2011;342:d1110.

Ker K, Edwards P, Perel P, Shakur H, Roberts I. Effect of tranexamic acid on surgical bleeding: systematic review and cumulative meta-analysis. *BMJ* 2012;344:e3054.

Law MR, Morris JK, Wald NJ. Use of blood pressure lowering drugs in the prevention of cardiovascular disease: meta-analysis of 147 randomised trials in the context of expectations from prospective epidemiological studies. *BMJ* 2009;338:b1665.

Leucht S, Corves C, Arbter D, Engel RR, Li C, Davis JM. Second-generation versus first-generation antipsychotic drugs for schizophrenia: a meta-analysis. *The Lancet* 2009;373(9657):31-41.

Leucht S, Tardy M, Komossa K et al. Antipsychotic drugs versus placebo for relapse prevention in schizophrenia: a systematic review and meta-analysis. *The Lancet* 2012;379(9831):2063-2071.

Tricco AC, Ivers NM, Grimshaw JM et al. Effectiveness of quality improvement strategies on the management of diabetes: a systematic review and meta-analysis. *The Lancet* 1916;379(9833):2252-2261.

Meta-analyses of observational studies:

Botteri E I. Smoking and colorectal cancer: A meta-analysis. *JAMA* 2008;300(23):2765-2778.

Clarke R, Bennett DA, Parish S et al. Homocysteine and Coronary Heart Disease: Meta-analysis of MTHFR Case-Control Studies, Avoiding Publication Bias. *PLoS Med* 2012;9(2):e1001177.

de Boer A, Taskila T, Ojajarvi A, Van Dijk F, Verbeek J. Cancer survivors and unemployment: A meta-analysis and meta-regression. *JAMA* 2009;301(7):753-762.

Hemingway H, Philipson P, Chen R et al. Evaluating the Quality of Research into a Single Prognostic Biomarker: A Systematic Review and Meta-analysis of 83 Studies of C-Reactive Protein in Stable Coronary Artery Disease. *PLoS Med* 2010;7(6):e1000286.

Norman RE, Byambaa M, De R, Butchart A, Scott J, Vos T. The Long-Term Health Consequences of Child Physical Abuse, Emotional Abuse, and Neglect: A Systematic Review and Meta-Analysis. *PLoS Med* 2012;9(11):e1001349.

Palomaki G, Melillo S, Bradley L. Association between 9p21 genomic markers and heart disease: A meta-analysis. *JAMA* 2010;303(7):648-656.

Pan A. Depression and risk of stroke morbidity and mortality: A meta-analysis and systematic review. *JAMA* 2011;306(11):1241-1249.

Renehan AG, Tyson M, Egger M, Heller RF, Zwahlen M. Body-mass index and incidence of cancer: a systematic review and meta-analysis of prospective observational studies. *The Lancet* 2008;371(9612):569-578.

Ronksley P, Brien S, Turner B, Mukamal K, Ghali W. Association of alcohol consumption with selected cardiovascular disease outcomes: a systematic review and meta-analysis. *BMJ* 2011;342:d671.

Ziegelbauer K, Speich B, Mausezahl D, Bos R, Keiser J, Utzinger J. Effect of Sanitation on Soil-Transmitted Helminth Infection: Systematic Review and Meta-Analysis. *PLoS Med* 2012;9(1):e1001162.

Meta-analyses of interventional studies

Brien S, Ronksley P, Turner B, Mukamal K, Ghali W. Effect of alcohol consumption on biological markers associated with risk of coronary heart disease: systematic review and meta-analysis of interventional studies. *BMJ* 2011;342:d636.
