# Supplementary figures and images for: Publication Bias in Recent Meta-Analyses
Source: PLoS One. 2013 Nov 27;8(11):e81823. doi: 10.1371/journal.pone.0081823 (PMC3868709; doi:10.1371/journal.pone.0081823)

## APPENDIX 5: SENSITIVITY ANALYSIS FOR META-ANALYSES OF CLINICAL TRIALS

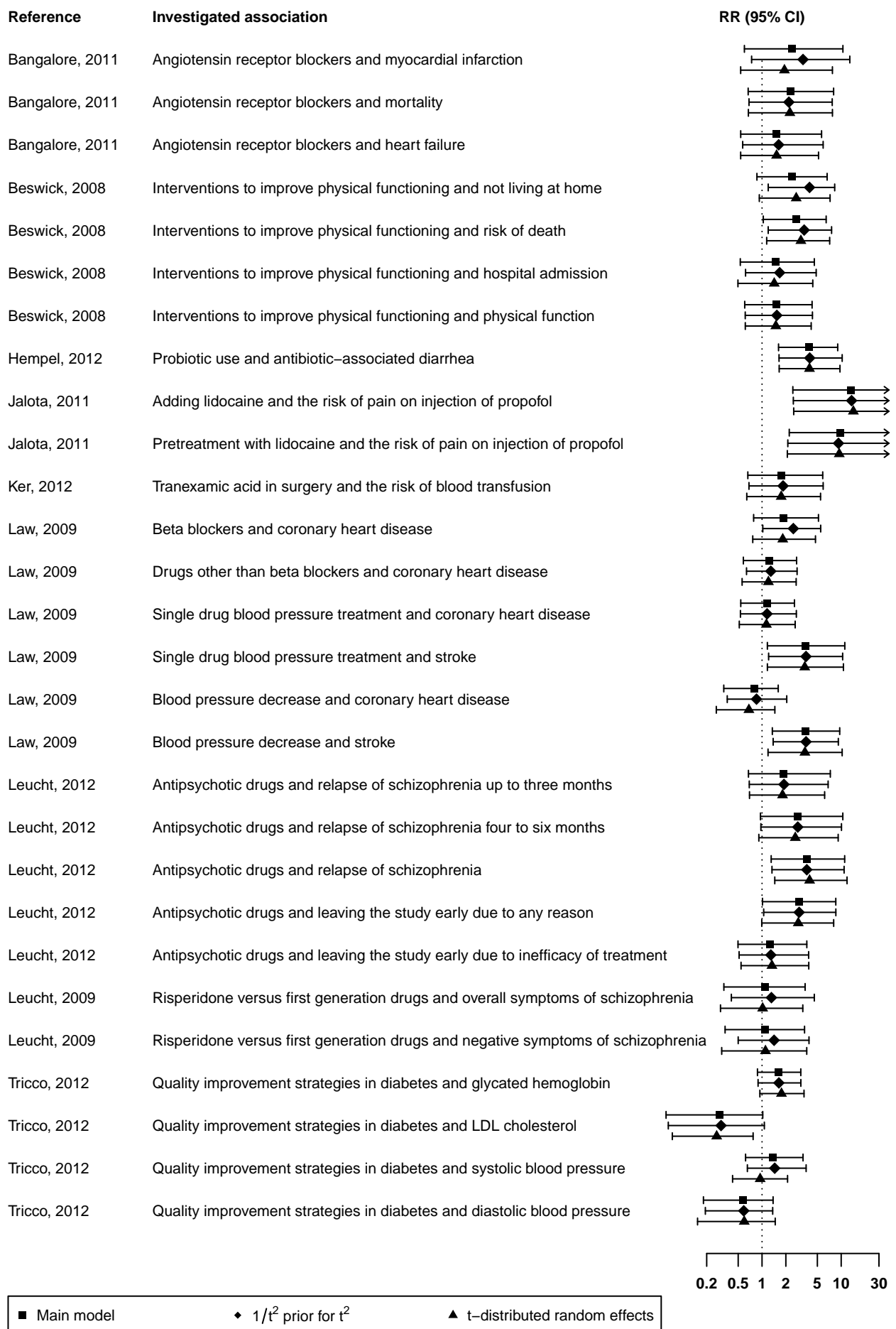

Supplement: Appendix S5 — Sensitivity analysis for meta-analyses of clinical trials. (PDF) [file pone.0081823.s005.pdf]

## APPENDIX 6: SENSITIVITY ANALYSIS FOR META-ANALYSES OF OBSERVATIONAL STUDIES

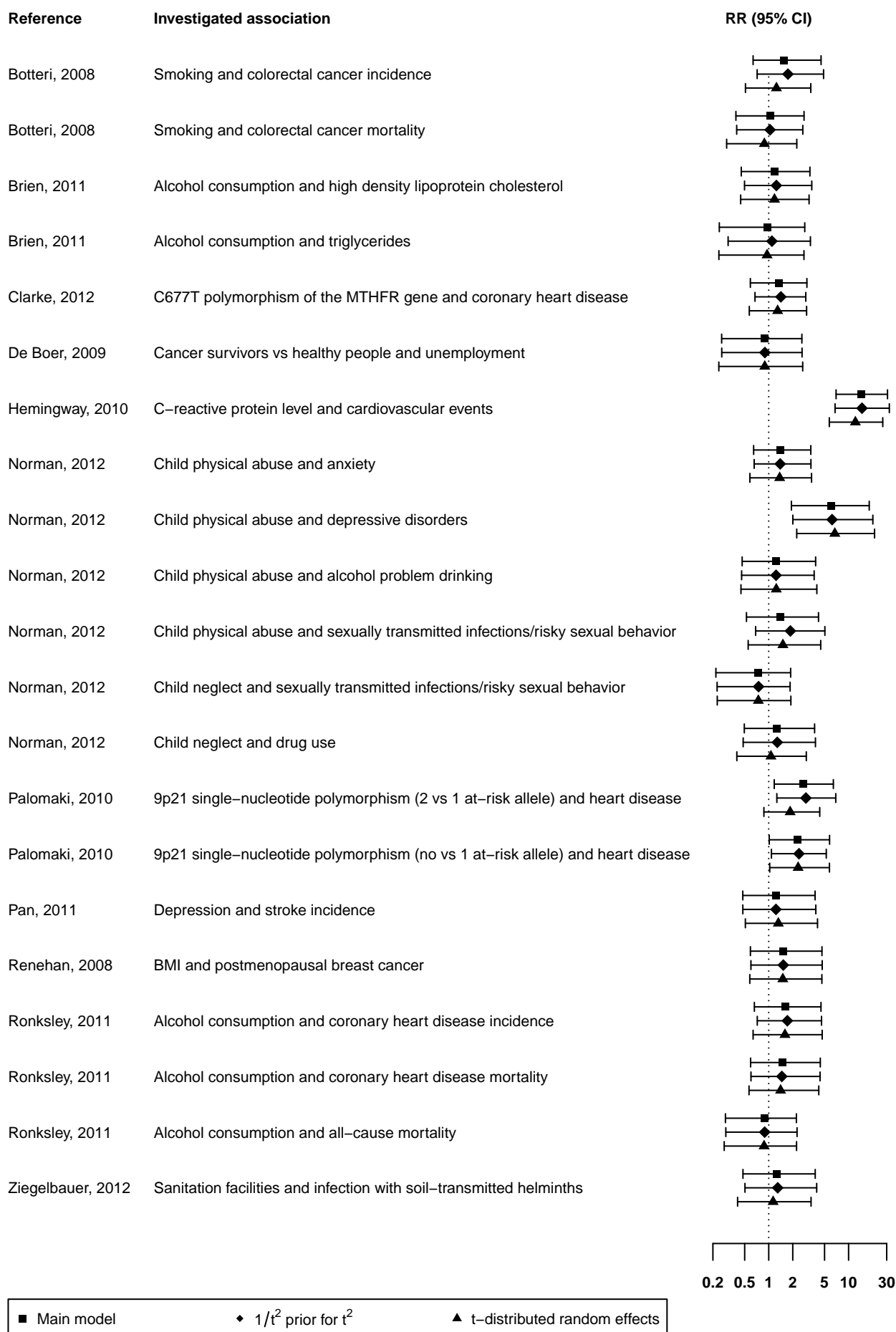

Supplement: Appendix S6 — Sensitivity analysis for meta-analyses of observational and interventional studies. (PDF) [file pone.0081823.s006.pdf]
